# Plant metabolites and functional foods in metastatic breast cancer: a supportive strategy for management

**DOI:** 10.3389/fphar.2025.1631232

**Published:** 2025-07-23

**Authors:** Pritya Jha, Varisha Anjum, Ahmed Adnan AL.-Khafagi, Sweta Joshi, Ammar Kadi, Areefa Anjum, Kamran Javed Naquvi, Irina Potoroko

**Affiliations:** ^1^ Department of Pharmacy, Banasthali Vidyapeeth, Banasthali, Rajasthan, India; ^2^ Department of Food and Biotechnology, South Ural State University, Chelyabinsk, Russia; ^3^ Anesthesia Techniques Department, College of Health and Medical Techniques, Al-Mustaqbal University, Babylon, Iraq; ^4^ Department of Food Technology, School of Interdisciplinary Science and Technology, Jamia Hamdard, New Delhi, India; ^5^ Centre for Interdisciplinary Research in Basic Sciences (CRIBSC), Jamia Millia Islamia, New Delhi, India; ^6^ Pharmacy Department, Tishk International University, Erbil, Iraq

**Keywords:** triple-negative breast cancer (TNBC), flavonoids, inflammation, oxidative stress, epiberberine, evodiamine, apoptosis, metastatic breast cancer

## Abstract

Breast cancer (BC) remains one of the leading causes of cancer-related mortality worldwide, with metastatic and triple-negative breast cancer (TNBC) subtypes presenting particular therapeutic challenges. This review critically explores the potential supportive role of plant-derived bioactive compounds present in functional foods and nutraceuticals in modulating cancer-related biological pathways. Metabolites such as flavonoids, alkaloids, terpenoids, and polyphenols have demonstrated anti-inflammatory, antioxidant, and pro-apoptotic effects in preclinical *in vitro* and *in vivo* studies. Specific compounds such as epiberberine, crocin, evodiamine, and extracts from Halodule uninervis have shown promising effects in limiting cancer cell invasion, proliferation, and angiogenesis. Advances in delivery technologies, including nanoformulations, may further enhance their bioavailability and targeted action. However, these findings are predominantly based on preclinical data, and rigorous *in vivo* validation and clinical trials are required to assess their translational potential. This review outlines emerging research directions and discusses how plant-derived compounds may contribute to integrated, evidence-based strategies for cancer care, particularly as adjuncts to conventional therapies rather than standalone treatments.

## 1 Introduction

Globally, 1 in 20 women will receive BC diagnosis at some point in their lives, and if current trends continue, there will be 3.2 million new instances of BC and 1.1 million deaths from the disease year by 2050. It is the second most common cancer globally and the most prevalent among women, with incidence rising rapidly, BC is increasing quickly. An estimated 310,720 cases of invasive BC are expected to occur in India in 2024. Women under 50 account for sixteen percent (16%) of these, India reports the highest number of BC-related deaths globally and has one of the lowest survival rates. Early detection can avert many fatalities in BC. However, detection is frequently fatal and delayed (Kim et al., 2025). Molecular targeted therapy, chemotherapy, radiation therapy, and surgery are currently used to treat BC. Even while surgery is still the main and most successful treatment for BC, it damages the patient’s organs irreversibly, which can be stressful and financially challenging (Qian et al., 2021; Salerno et al., 2018; Tada et al., 2023). Alternative medical treatments like endocrine therapy, molecular targeted therapy, and chemotherapy may cause drug-induced liver damage, which lowers survival and quality of life for patients. Therefore, finding novel treatments for BC is crucial (Bae et al., 2015).

The treatments are frequently influenced by the subtypes, namely, hormone receptor positive, Her two amplified, and triple-negative. The recommendation for surgery has grown, particularly in cases of metastasis (Peart, 2015; Tommasi et al., 2024). The size of the initial breast tumor, involvement of axillary lymph nodes, and distant metastases all affect the stage of BC, which is also a predictor of survival (Prat et al., 2015). A multimodal and multidisciplinary approach incorporating natural plant products has emerged to improve survival outcomes in response to this variability in disease presentation and treatment response. Early identification and disease diagnosis remain crucial for these multimodal techniques (Wörmann, 2017). One of the biggest obstacles to the effective treatment of many cancer types is the possibility that tumors could develop multidrug resistance (MDR), or resistance to chemotherapy. A process known as multidrug resistance occurs when cancer cells that were initially susceptible to a single anti-cancer medication eventually develop resistance to several unrelated medications that differ physically, functionally, and possibly molecularly (Emran et al., 2022). Cancer cells may exhibit acquired MDR, a more problematic form of MDR that results in chemotherapy failure even after dose escalation to toxic levels, or they may exhibit intrinsic MDR, which is caused by explicit nature or other genetic characteristics of the cells. When cancer cells with inherent MDR are first exposed to an anticancer drug, they show resistance to chemotherapy (Kartal-Yandim et al., 2016).

As anti-tumor and anti-cancer medicines, natural plant metabolites have demonstrated encouraging outcomes. They have shown reduced toxicity and fewer instances of acquired resistance to hormonally targeted anticancer drugs (multidrug resistances, as observed with various anti-cancer agents). These effects stem from their immunomodulatory, anti-inflammatory, and antioxidant properties, as well as their ability to regulate the growth and death of malignant cells (Bonofiglio et al., 2016). This is done in a way that presents a chemo-preventative feature that is safe for long-term use and can be prophylactic (Nguyen et al., 2019). Flavonoids, alkaloids, terpenoids, and coumarins are metabolites of natural plant products that are well-known for their anti-inflammatory and antioxidant qualities (glabridin, curcumin, arctigenin, and ajoene) as well as their ability to activate lymphocytes (quinic acid, β-carotene, epigallocatechin-3-gallate, and ginsan). These powerful immunomodulatory qualities are necessary to inhibit or combat cancer cells (Baraya et al., 2017). Bioactive substances that can function as endocrine disruptors for hormonal disorders—which are frequently the cause of these cancer outcomes include phytoestrogens (non-steroidal phenolic metabolites having structural similarities comparable to steroids like oestrogen) and isoflavinoids. It has been observed that certain plant flavonoids have chemopreventative, estrogenic, and/or anti-estrogenic qualities (Dietz et al., 2016; Křížová et al., 2019). In addition to their capacity to cause oxidative stress and cancer induction through oestrogen receptor signaling, they can also inhibit oestrogen receptor dependent (cell growth and proliferations) and independent (free radical generation and genotoxic agents) connections (Bak et al., 2016). The purpose of this review is to objectively assess natural bioactive chemicals’ potential as supportive medicines in the treatment of BC. It examines their significance in treating MDR and highlights their suggested modes of action, which include pro-apoptotic, antioxidant, and anti-inflammatory actions. The review addresses how these chemicals may function as adjuncts that could improve therapy tolerability and patient quality of life, rather than offering them as substitutes for traditional medicines. Additionally covered are the categorization of bioactive metabolites produced from plants, noteworthy preclinical results, and their applicability to different subtypes of BC. The review also assesses their ability to lessen side effects and resistance linked to chemotherapy. Future directions for incorporating these substances into evidence-based cancer therapy are examined, along with a summary of recent preclinical and emerging clinical investigations.

## 2 Molecular mechanisms driving TNBC progression and potential therapeutic targets

**FIGURE 1:**
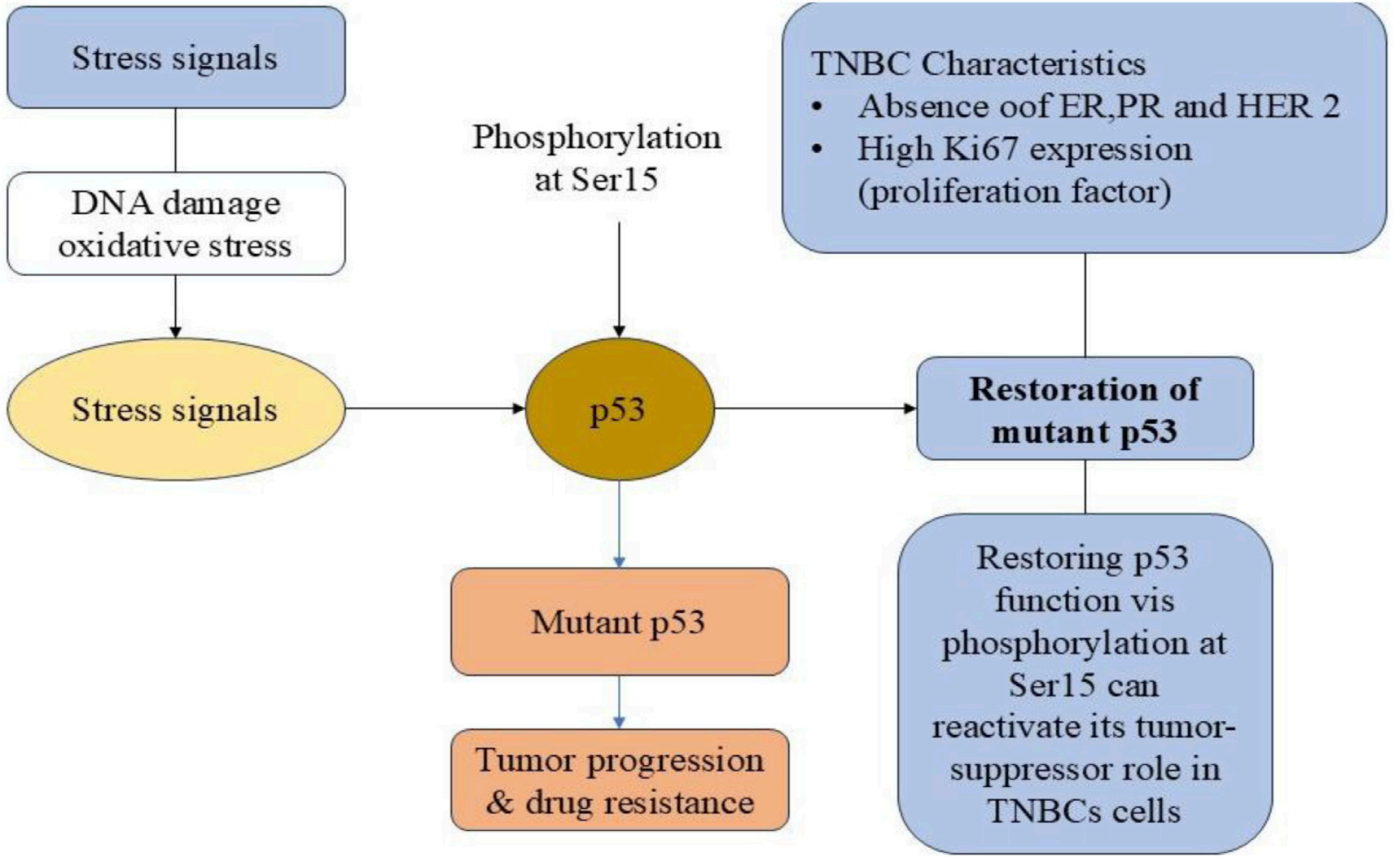
Molecular mechanisms contributing to the progression of triple-negative breast cancer (TNBC).

TNBC is characterized by its aggressive nature, high metastatic potential and poor prognosis due to the absence of hormone receptors and HER2 amplification. The lack of specific molecular targets makes conventional treatment options, such as chemotherapy, less effective, often leading to drug resistance and tumor recurrence. Understanding the molecular mechanisms underlying TNBC progression is crucial for identifying novel therapeutic targets. TNBC has a high expression of the proliferation marker Ki67, which is linked to its aggressive pathological features (Davey et al., 2021). By controlling a number of cellular functions, including as cell cycle progression, apoptosis, and cellular response to stress, p38 is recognized to be essential for preserving cellular homeostasis (Martínez-Limón et al., 2020). The tumor suppressor protein p53 may also be activated by p38. Known as the “guardian of the genome,” p53 is essential for controlling DNA repair, cell cycle progression, and apoptosis in response to cellular stress (Marvalim et al., 2023). About 80% of TNBC cases include p53 gene mutations, which are linked to numerous malignancies and increase tumor aggressiveness and treatment resistance (Berke et al., 2022). Therefore, one intriguing approach in the creation of TNBC-targeted treatments is to target the mutant p53 and restore its wild-type form (Figure 1). One crucial post-translational alteration that controls p53 stability and activity is phosphorylation of p53 at ser15 (Hientz et al., 2017). Furthermore, research has demonstrated that in TNBC, phosphorylating mutant p53 at ser15 restores its tumor-suppressor function (Mesmar et al., 2022; Perdrix et al., 2017; Synnott et al., 2020). A key tactic in cancer treatment is to induce apoptosis in cancer cells in addition to preventing unchecked growth (Carneiro and El-Deiry, 2020). TNBC and other cancer cells frequently avoid apoptosis, which promotes tumor growth and treatment failure (Alsamri et al., 2023; Kamalabadi-Farahani et al., 2019).

**FIGURE 2:**
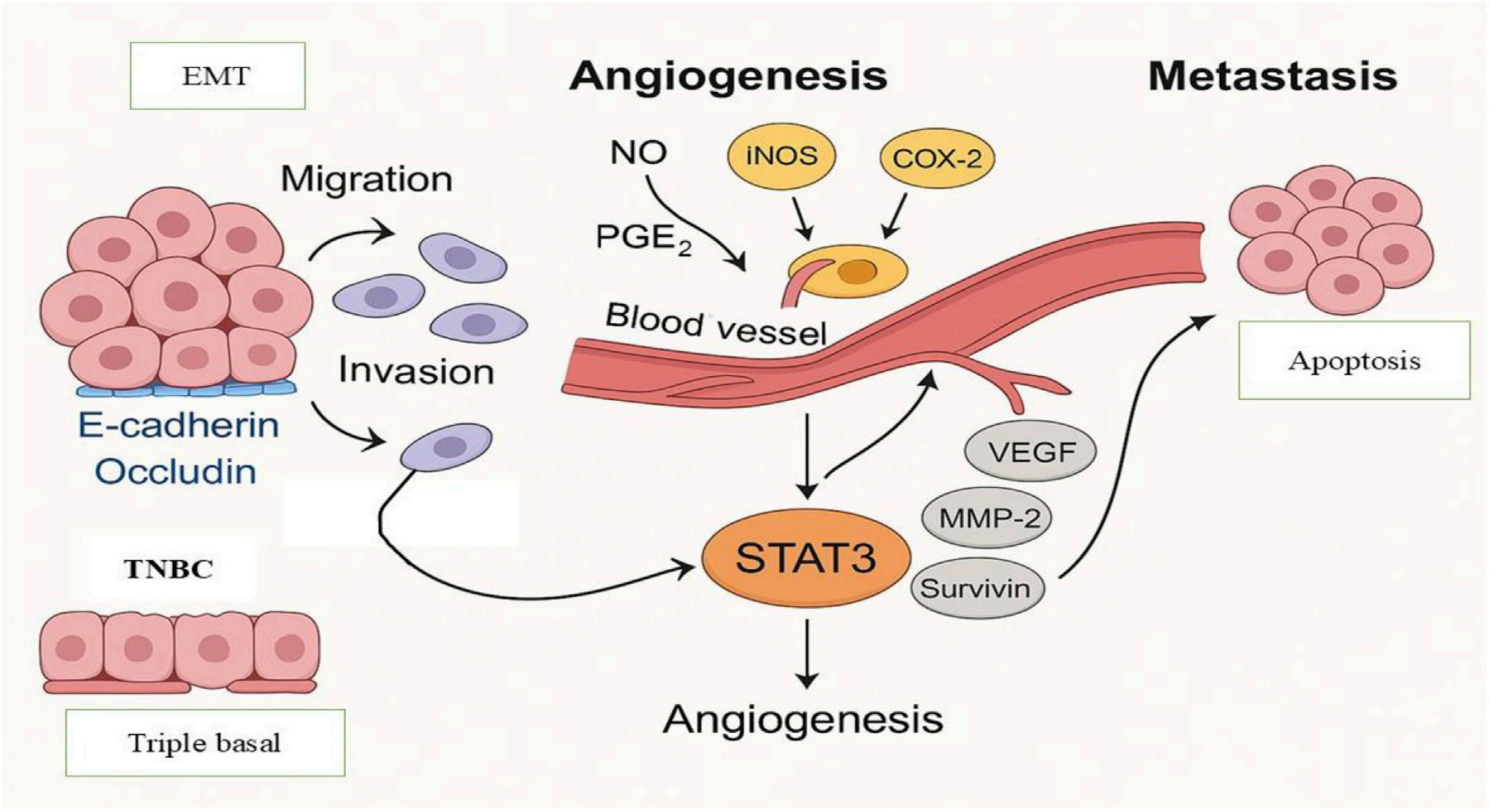
Molecular Mechanisms Driving Metastasis and Angiogenesis in TNBC. The functions of the EMT, loss of E-cadherin and occludin, and the angiogenic activity triggered by COX-2, iNOS, NO, and PGE2 are highlights of important molecular pathways implicated in metastasis and angiogenesis in triple-negative breast cancer (TNBC). The STAT3-mediated transcription of genes that support cell motility, invasion, proliferation, and survival highlighted in the diagram.

Thus, triggering apoptotic pathways turns out to be a powerful cancer therapeutic strategy. This is especially relevant, as many conventional anticancer drugs induce apoptosis through excessive ROS generation. Nonetheless, a number of anticancer medications have been reported to work by causing ROS-independent apoptosis in cancer cells with little adverse effects on healthy cells (Ivanova et al., 2016). Metastasis refers to the complex process by which cancer cells spread from the primary tumor site to distant organs. It contributes to the development of cancer and accounts for a significant percentage of cancer-related fatalities. Because of its aggressive nature, poor prognosis, and resistance to treatment, TNBC metastasis poses a serious clinical problem (Janiszewska et al., 2020). Two important elements of cancer metastasis are cell invasion and migration. In order to prevent metastatic spread and overcome treatment resistance, it is imperative to target their underlying mechanisms. Increased migration and invasion of cancer cells are caused by metalloproteinases (MMPs) breaking down the extracellular matrix (ECM) and dysregulating cell-cell adhesion (Gonzalez-Avila et al., 2019). The contact between cancer cells and the ECM, which mediates several stages of the metastatic cascade, is also essential for cancer spread (Eble and Niland, 2019). By interacting with several ECM metabolites, such as collagen, fibronectin, and laminin, cancer cells separate from the original tumor site and spread across the surrounding ECM (Walker et al., 2018). One of the key processes causing TNBC tumor metastasis has been identified as the (EMT) (Grasset et al., 2022; Jang et al., 2016). Cell migration and invasion increase and cell–cell contacts decrease during EMT (Brabletz et al., 2018). Increased tumor aggressiveness in individuals with BC has been associated with the deregulation of important molecular factors, such as the cell adhesion molecule E-cadherin and the tight junction-forming protein occluding as shown in Figure 2 (Padmanaban et al., 2019; Rubtsova et al., 2022).

Tumor growth, invasion, and metastasis are all significantly influenced by angiogenesis, the process of creating new blood vessels (Lugano et al., 2020). TNBC tumors in particular are frequently very angiogenic, which promotes tumor growth and the spread of metastases (Ribatti et al., 2016). Thus, blocking angiogenesis by focusing on pro-angiogenic factors has become a viable treatment approach to stop the growth and metastasis of TNBC tumors. Angiogenesis in TNBC has been linked to both cyclooxygenase-2 (COX-2) and inducible nitric oxide synthase (iNOS) (Granados-Principal et al., 2015; Pan et al., 2020). Nitric oxide (NO) and prostaglandin E2 (PGE2) are produced by the enzymes iNOS and COX-2, respectively, in response to external stimuli found in the tumor microenvironment. Strong angiogenesis mediators, NO and PGE2 are upregulated in TNBC, which encourages metastasis (Basudhar et al., 2017). Over the past few decades, plant-derived dietary metabolites have attracted increasing attention in cancer research, especially in conjunction with the resurgence of interest in traditional medicine. Low toxicity, wide availability, and typically positive tolerance profiles are some of its appealing qualities (Sferrazza et al., 2020).

## 3 Plant metabolites in metastatic breast cancer therapy

Plant extracts and their derivatives have been utilized in traditional medicine practices since ancient civilizations to treat a wide range of ailments, such as diabetes, arthritis, tuberclosis, malaria, and dermatological issues (Prasathkumar et al., 2021). The abundance of secondary metabolites found in plants makes them a rich source of therapeutic substances. These secondary metabolites have strong medicinal qualities, such as anti-inflammatory, anti-cancer, antibacterial, and antioxidant qualities, in addition to their ecological function. Plant-derived chemicals are becoming more and more popular as anticancer treatments because of their low toxicity, chemical variety, and natural origin (Roy et al., 2022). Actually, out of the 247 anticancer medications that have been approved in the past 40 years, only 29 are entirely synthetic. With an estimated 10 million deaths in 2020, cancer remains one of the leading global health threats and a major cause of mortality (Min and Lee, 2022). Specifically, BC is the most common, accounting for the fourth most common cause of cancer-related deaths globally (Bray et al., 2024; Min and Lee, 2022). The development of different therapeutic options for BC has advanced significantly over time. Radiation therapy, chemotherapy, and surgery are a few of them (Wang and Wu, 2023). Although the goal of these treatments is to eradicate cancer cells, they frequently cause a variety of adverse effects, such as nausea, vomiting, inflammation, hormone abnormalities, and harm to healthy tissues due to their non-selective nature. On the other hand, targeted and hormonal medicines provide targeted treatment with fewer adverse effects. Targeted therapy works well against the human epidermal growth factor 2 receptor, whereas hormonal therapy targets the estrogen and progesterone receptors. These three receptors are absent in TNBC, which makes up 15%–20% of all cases of BC and is resistant to these treatments (Anand et al., 2023). TNBC is the most aggressive subtype of BC and is therefore linked to the worst prognosis (Bao and Prasad, 2019). As a result, there is a growing need for alternative therapeutic approaches, with researchers exploring a wide range of supportive interventions derived from plants (Table 1).

**TABLE 1:** Summary of key plant-derived metabolites evaluated for breast cancer activity.

Plant metabolites	Source	Reported action	References
Epiberberine	*Coptis chinensis*	Inhibits STAT3, induces apoptosis, suppresses migration and invasion	Zou et al. (2016)
Crocin	*Crocus sativus*	Inhibits Wnt/β-catenin, induces caspase-3, suppresses migration	Arzi et al. (2018a)
Halodule Extract	*Halodule uninervis*	Downregulates STAT3 and COX-2, induces apoptosis	Wehbe et al. (2024), Wehbe et al. (2025)
Ziziphus spp.	*Z. nummularia*, *Z. spina-christi*	Induces apoptosis, cell cycle arrest	(El Maaiden et al., 2020; Farmani et al., 2016; Golla, 2018; Mesmar et al., 2021)
Evodiamine	*Evodia rutaecarpa*	Induces ROS-mediated apoptosis, inhibits migration	Liu et al. (2020)
Rutaecarpine	*Evodia rutaecarpa*	Induces apoptosis *in vitro*	Wang et al. (2022)
Icaritin	*Epimedium* spp.	Induces apoptosis, modulates lipid metabolism, radiosensitizer	Du et al. (2020)
Matrine	*Sophora flavescens*	Inhibits PI3K/AKT/mTOR, induces apoptosis, activates AMPK	Du et al. (2020)
Shikonin	*Lithospermum erythrorhizon*	Induces apoptosis, ROS generation, ferroptosis	Chen et al. (2019)
Osthole	*Cnidium monnieri*	Inhibits FAK/Src/Rac1, modulates HDAC, anti-angiogenic, synergistic with PTX	Yang et al. (2013)
Arctigenin	*Arctium lappa*	Anti-EMT, enhanced bioavailability via nanoliposomes	Feng et al. (2017)

## 4 Anti-metastatic effects of plant metabolites

### 4.1 Epiberberine

Natural products are increasingly being researched for their potential to treat a variety of ailments, including cancer, and have long served as a basis for contemporary medication discovery. These substances are important leads in pharmacological research since they affect a variety of biological pathways. The possible anti-cancer effects of Coptidis rhizoma (Huanglian), which has long been used in Chinese medicine to treat inflammation and detoxification, have been studied. Epiberberine (EPI), one of its main alkaloids, has a variety of pharmacological properties, such as anti-inflammatory, anti-adipogenic, and anti-dyslipidemic actions. Although preclinical research has demonstrated that EPI inhibits cancer cell lines like HCT116 and MCF7, its precise mechanisms in BC, especially in relation to metastasis-induced osteolysis, are still poorly understood (Wei et al., 2024). Natural products have attracted a lot of interest as possible, EPIs with a wide range of pharmacological effects and are essential resources for contemporary drug research. EPI has shown to have an anti-adipogenic effect on the differentiation of 3T3-L1 adipocytes (Choi et al., 2015). Furthermore, (Zou et al., 2016), discovered that EPI has anti-dyslipidemia properties and decreased HMGCR mRNA and protein expressions. EPI has not thoroughly investigated, despite earlier research showing that it inhibits HCT116 and MCF7 cells (Tan et al., 2017). The pharmacological properties of EPI, a berberine isomer, include anti-inflammatory, anti-cancer, anti-dyslipidemia, anti-bacterial, anti-adipogenesis, and anti-alzheimer’s disease (Li et al., 2015; Wang Y. et al., 2020; Yu C. et al., 2020). *In vitro* studies suggest that EPI may induce apoptosis and inhibit migration and invasion in BC cell lines like MDA-MB-231 and MCF-7 (Wang Z. et al., 2020; Bolz et al., 2021; Wang Y. et al., 2020). In one study, MDA-MB-231 cells were exposed to different EPI doses (1–40 µM) for 48 h, which resulted in enhanced apoptosis and dose-dependent inhibition of migration. The negative control in this experiment was DMSO (Xu S. et al., 2021). EPI’s effects on BC cells included reversing the EMT process and stimulating the Wnt/β-Catenin signaling pathway (Wei et al., 2024). According to Wei et al.'s research, EPI may prevent osteoclast formation and function, which would lessen bone loss brought on by BC cells (Dian et al., 2022). Additionally, it has been identified as a potential PAINS (Pan-Assay Interference Compounds) molecule, highlighting the need for additional mechanistic analysis and cautious interpretation (Bolz et al., 2021).

### 4.2 Halodule uninervis extract

Numerous bioactive metabolites found in nature have the potential to be developed into new medicinal medicines. Particularly, plants have been utilized for ages in traditional medicine and continue to be a widely available and culturally relevant source of treatment for a wide range of acute and chronic ailments. Around 80% of people worldwide use plant-based or botanical medications for therapeutic purposes, according to the World Health Organization (von Schoen-Angerer et al., 2023). The marine environment, which comprises approximately 95% of the biosphere, is becoming more and more acknowledged as a valuable and biodiverse source for drug development, in addition to terrestrial ecosystems (Danovaro et al., 2017).

Seagrass is a particularly notable marine plant. They have crucial ecological roles by stabilizing sediments, promoting marine biodiversity, and enhancing coastal food security because they are the only blooming plants that are suited to completely submerged marine habitats. Seagrasses have been utilized in traditional medicine to treat skin conditions, fever, muscle soreness, and wounds (Ameen et al., 2024). One noteworthy species is Halodule uninervis (HUE), a member of the Cymodoceaceae family that can withstand stress and is well-known for having a high concentration of secondary metabolites, including flavonoids, phenols, and tannins, all of which enhance its medicinal potential. According to reports, H. uninervis extracts have antibacterial (Supriadi et al., 2016), antioxidant (Ghandourah et al., 2021), and anticancer (Parthasarathi et al., 2021).

**FIGURE 3:**
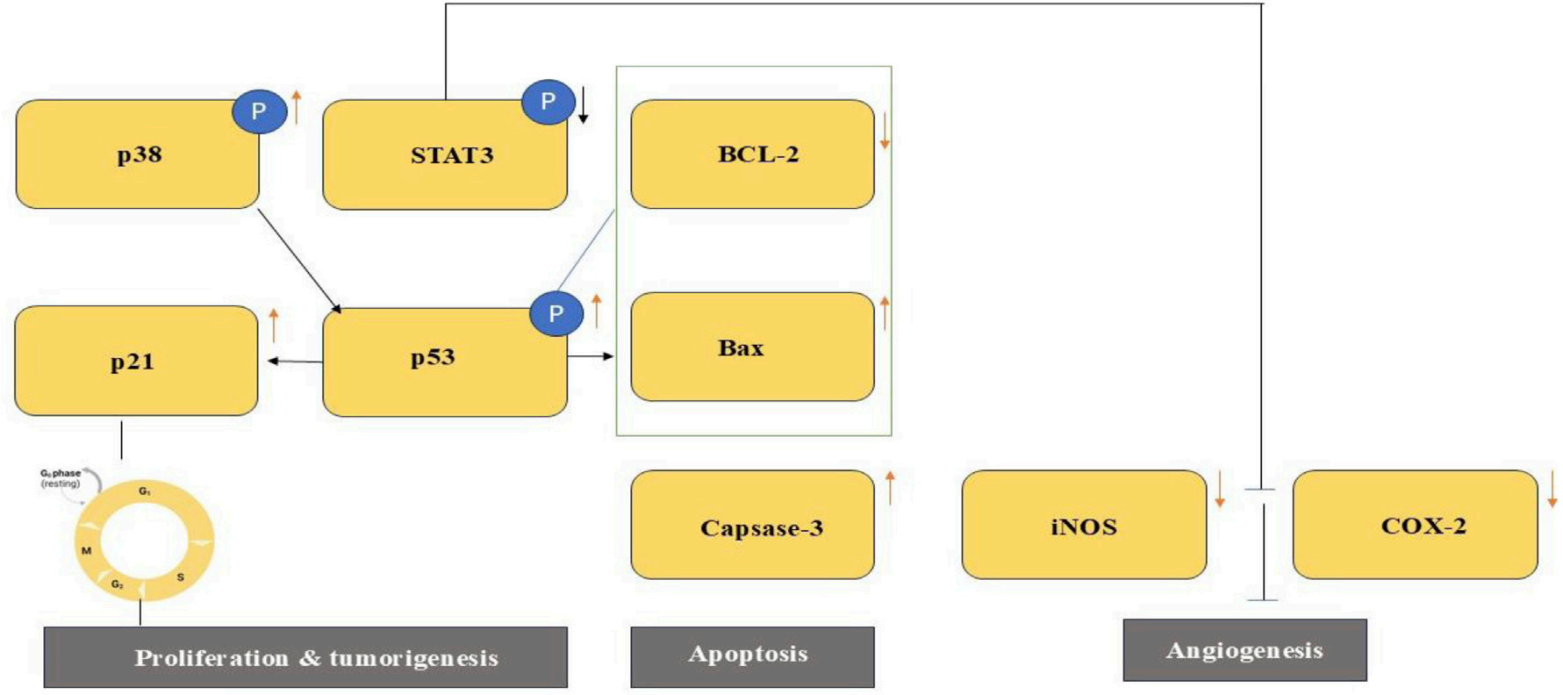
An illustration of the Halodule uninervis ethanolic extract’s suggested anti-malignant actions in MDA-MB-231 cells. HUE may have done this by specifically targeting STAT3 and related proteins, which prevented the hallmarks of cancer.

According to recent research, H. uninervis’s ethanolic extract has potent antioxidant properties and may affect the behavior of TNBC cells by focusing on a number of cancer-related pathways (Wehbe et al., 2024). The extract has been demonstrated to prevent the growth of cancer cells *in vitro* by causing intrinsic apoptosis and cell cycle arrest. It also hindered angiogenesis, adhesion, invasion, and cellular motility effects that may have been caused by blocking the constitutively active STAT3 signaling pathway. A combined anti-inflammatory and anti-angiogenic impact are suggested by the fact that H. uninervis extract decreased the production of COX-2, a crucial enzyme involved in inflammation and angiogenesis. The ethnopharmacological significance of researching crude plant extracts is highlighted by the possibility that the complete extract exhibits synergistic activity that outweighs the impact of isolated components. Under experimental settings, HUE was given to MDA-MB-231 cells at doses between 25 and 200 μg/mL for a duration of 24 h. This led to the downregulation of the expression of COX-2 and STAT3. The vehicle control was methanol. Nevertheless, these effects have not yet been confirmed *in vivo* (Wehbe et al., 2024). Apart from its direct biological activity, H. uninervis has demonstrated potential in the field of green nanotechnology. Gold nanoparticles (AuNPs), which are renowned for their biocompatibility and adjustable anticancer capabilities, have been biosynthesised from the plant. Cost-effectiveness, safety, scalability, and ecological sustainability are some of the benefits of plant-mediated synthesis (Wehbe et al., 2025). Here, ecologically safe AuNPs with early indications of anticancer activity were created using H. uninervis. Because of their nontoxicity, biodegradability, and encouraging bioactivity, these nanoparticles may be able to supplement or improve on current cancer treatments. Furthermore, when combined with traditional therapies, surface functionalization of these AuNPs with chemotherapeutic drugs or specific ligands may enhance selectivity and intracellular absorption, thus producing synergistic anticancer effects (Figure 3).

### 4.3 Saffron

Crocin and crocetin, two bioactive carotenoids found in saffron, a traditional medicinal spice made from the dried stigma of Crocus sativus L., have shown antiproliferative and pro-apoptotic effects in a number of preclinical investigations. The major ingredient responsible for the spice’s anti-cancer and anti-metastatic properties has been found to be crocin (Dariushnejad et al., 2020; Ghorbanzadeh et al., 2017). Crocin has been demonstrated *in vitro* to cause apoptosis, decrease cell motility, and prevent invasion in MDA-MB-231 TNBC cells. However, clinical and thorough *in vivo* validation of these benefits is still pending (Arzi et al., 2018b). According to Ghorbanzadeh et al. (2017), crocin seems to work mechanistically by interfering with mitotic progression in BC cells via altering microtubule dynamics. In mice models of breast tumors produced by N-nitroso-N-methylurea, it has also demonstrated antiproliferative and pro-apoptotic activity against a variety of cancer cell lines, including HCT-116, SW-480, HT-29, AGS, HL-60, HeLa, MCF-7, BxPC-3, and Tca8113. According to the suggested mechanism, the cell cycle is arrested at both the G2/M and G1 phases, and apoptotic pathways are activated through elevated Bax/Bcl-2 ratios (Fakhri et al., 2022; Chryssanthi et al., 2007). Crocin significantly increased caspase-3 activity and cytotoxicity in one study when it was given to MDA-MB-231 cells at doses between 50 and 200 µM for 48 h. Mice with xenograft tumors received an intraperitoneal dose of 50 mg/kg of crocin for 21 days, which inhibited the tumor. Nevertheless, human clinical trials are still needed to validate these encouraging findings (Ekem et al., 2020). According to Razmaraii et al. (2016) and Vali et al. (2015), crocin and crocetin have also been shown to improve the effectiveness of traditional chemotherapeutics, including doxorubicin, paclitaxel, pemetrexed, and cisplatin, when taken together in preclinical animals. Nevertheless, no research has yet to effectively illustrate the combined anti-metastatic synergistic effects of crocin and crocetin in a clinical or advanced preclinical setting.

### 4.4 Ziziphus

Organic metabolites that are found naturally in plants and are known for a variety of biological actions are called phytochemicals. Many plant species contain compounds like alkaloids and flavonoids, which are becoming more and more known for their possible anticancer effects. These plant metabolites usually contain a variety of functional groups in their core structure, which adds to their wide range of pharmacological actions (Bhambhani et al., 2021). Many cultures in China, India, Pakistan, and the Middle East have long utilized the genus Ziziphus (family Rhamnaceae), which includes more than 58 known species of small trees and prickly bushes that are mostly found in dry and semi-arid areas, in traditional medicine. The medicinal potential, nutritional worth, and other health-promoting benefits of these species make them valuable (El Maaiden et al., 2020; Golla, 2018). Ziziphus has anti-inflammatory, gastroprotective, antidiabetic, neuroprotective, and dermatological benefits, including anti-aging, wound healing, and sunburn relief. Early research points to potential anti-infective and anticancer qualities, despite the fact that scientific knowledge of its mechanisms is still restricted.

According to phytochemical studies, Ziziphus species are abundant in bioactive substances, such as alkaloids (particularly cyclopeptide alkaloids), flavonoids, saponins, terpenoids, and trace amounts of cholinergic acids, steroids, cerebrosides, aromatic or polyaromatic compounds, and nucleosides. These compounds total more than 431 different metabolites (Alla et al., 2025). Ziziphus nummularia is one among these that has demonstrated significant cytotoxic activity *in vitro*, indicating broad-spectrum anticancer potential. Several cancer cell lines, such as colon adenocarcinoma (HT-29), breast cancer (MCF-7), ovarian cancer (OVCAR-3), leukemia (K-562), kidney carcinoma (A-498), and pancreatic cancer cells (Capan-2) have been shown to be inhibited by its extracts (Alghamdi et al., 2024; Ray and Dewanjee, 2015). In a similar vein, Ziziphus spina-christi, also referred to as Christ’s thorn jujube or Sidr, has long been used to treat pain and inflammation. Its anticancer potential has been investigated recently, and cytotoxic effects on HeLa, MDA-MB-468, and MCF-7 BC cell lines have been found. It has been observed that Z. spina-christi’s ethanolic extract causes MCF-7 cells to undergo apoptosis and G1/S phase cell cycle arrest. *In vitro* experiments with Z. nummularia methanolic extracts at concentrations between 10 and 100 μg/mL over a 48-h period showed that the IC_50_ values in MCF-7 and MDA-MB-468 cells were approximately 40 μg/mL. Paclitaxel or doxorubicin are commonly used as positive controls, while Z. spina-christi ethanolic extracts (25–150 μg/mL) likewise caused apoptotic effects and cell cycle arrest in MCF-7 cells (Farmani et al., 2016). These results demonstrate Ziziphus species’ phytochemical diversity and their anticancer properties. We examined the phytochemical makeup of Z. nummularia and asseseed it’s *in vitro* effects on TNBC, because of these studies as well as the paucity of research on the plant’s possible therapeutic qualities against BC.

## 5 Overcoming chemoresistance with natural substances

Cancer chemoresistance is a complex phenomenon characterized by multiple mechanisms, including drug efflux, apoptosis suppression, increased detoxification, DNA repair, modified drug targets, stem cell renewal, and epithelial-to-mesenchymal transition (Housman et al., 2014; Wu et al., 2014). BC continues to be the most often diagnosed malignancy and the primary cause of cancer-related mortality among women globally. About 25% of BC exhibit overexpression of the human epidermal growth factor receptor 2 (HER2), a marker linked to aggressive tumor characteristics and unfavorable prognosis. Despite the substantial enhancement in clinical outcomes provided by HER2-targeted therapies, especially HER2 tyrosine kinase inhibitors (HER2-TKIs) such as lapatinib, resistance to these treatments frequently emerges swiftly, usually within 1 year, thereby complicating the management of HER2-positive metastatic breast cancer (Łukasiewicz et al., 2021). Traditional Chinese Medicine (TCM), which combines herbal medicines with techniques such as acupuncture and moxibustion, has a longstanding history of treating chronic illnesses, including cancer. This legacy has stimulated research in utilizing natural compounds to combat chemoresistance. Wang et al. (2016) assessed the synergistic impact of doxorubicin and osthole on bladder cancer T24/ADM cells. Osthole exhibited minimal toxicity at concentrations up to 17 μM (IC_50_ = 76.5 μM); nevertheless, when co-administered at this concentration, it diminished the IC_50_ of doxorubicin from 1.0 to 0.4 μM, resulting in a 2.5-fold enhancement in drug sensitivity.

Recent research have examined Artemisia argyi, a TCM plant, for its potential to mitigate HER2-TKI resistance. Analysis of The Cancer Genome Atlas (TCGA) data indicated that diminished expression of transmembrane serine protease 2 (TMPRSS2), a membrane-bound proteolytic enzyme, correlated with unfavourable prognosis in BC. TMPRSS2 expression was significantly elevated in patients responsive to lapatinib, indicating its role in influencing medication sensitivity. Bioactive substances extracted from A. argyi, such as eriodictyol and umbelliferone, shown the ability to suppress the proliferation of lapatinib-resistant HER2-positive BC cells. Mechanistic studies revealed that these drugs enhanced TMPRSS2 expression, consequently inhibiting HER2 kinase activity and reinstating drug sensitivity. The findings underscore A. argyi as a potential adjuvant in addressing HER2-TKI resistance and identify TMPRSS2 as a prospective therapeutic target (Ho et al., 2024). Likewise, evodiamine—a bioactive alkaloid—demonstrates promise in addressing medication resistance. Evodiamine markedly increased drug sensitivity in cisplatin-resistant A549/DDP lung cancer cells. At a concentration of 0.25 μg/mL, it diminished the IC_50_ of cisplatin from 76.70 to 6.70 μg/mL, representing an over 11-fold enhancement in sensitivity, while maintaining cell viability in both resistant and sensitive cells at concentrations up to 5 μg/mL (Wang et al., 2015). Evodiamine was discovered to inhibit the expression of pIKKα, MDR1 mRNA, and the anti-apoptotic protein Bcl-2, indicating its potential to reverse resistance via several mechanisms. Celastrol, a natural chemical, demonstrates significant anti-drug resistance properties. In K562/A02 leukemia cells, celastrol markedly decreased MDR1 protein levels, augmented intracellular drug accumulation, and amplified chemosensitivity by as much as 117.9-fold. These studies jointly highlight the capacity of natural chemicals to influence treatment resistance in cancer cells. Nonetheless, the ability to consistently replicate these promising *in vitro* discoveries *in vivo* or in animal models remains a pivotal inquiry. Certain animal research have initiated the demonstration of these effects (Table 2), although additional thorough assessments are required to confirm their translational applicability in clinical environments.

**TABLE 2:** Plant metabolites that overcome drug resistance in animal models.

Plant metabolites/Extract	Cancer model	Treatment & Comparison	Key outcomes	References
Changweiqing Extract	HCT116/L-OHP (colon, xenograft in nude mice)	Oxaliplatin vs Oxaliplatin + Changweiqing (5x/week, 3tweeks)	Tumor size reduced 3.1× in experimental group	Komen et al. (2022)
Huatan Sanjie Formula	MCF-7/ADM (breast, xenograft)	Doxorubicin vs Dox + extract (2 doses: days 8 & 15)	4.8× tumor weight reduction; ↑ doxorubicin levels; ↓ MDR1 protein	Feng et al. (2014)
Epigallocatechin gallate	KBV200 (oral, xenograft)	Vincristine ± EGCG	↑ Vincristine effect 13×; ↓ MDR1 and LRP mRNA levels in tumor tissue	Almaguer et al., 2023
Puerarin + 5-Fluorouracil	(unspecified tumor model)	5-FU alone vs 5-FU + puerarin	Tumor inhibition ↑ from 18.1% to 56.7%; ↓ MDR1 and MRP protein levels	Wang et al. (2005)
Curcumin	HCT8/VCR (colon, xenograft)	Vincristine ± curcumin	↑ Chemo effect; ↓ MDR1 and survivin proteins in tumors	Lu et al. (2011)
Cepharanthine	EAC/ADR (ascites carcinoma)	Doxorubicin ± cepharanthine	↑ Cell sensitivity 13×; ↑ mouse lifespan 75.4%; ↓ NF-κB	Wang (2008)
Cepharanthine hydrochloride	Hca (liver cancer, drug-resistant)	Multiple chemo drugs ± cepharanthine hydrochloride	↓ MDR1 and MRP1; ↑ drug uptake; ↑ mouse survival	Wang (2008)
Matrine	S180 sarcoma (drug-resistant)	100 mg/kg/day × 10 days	↓ MDR1 (78%), LRP (84%), Topo II (65%) in tumor tissues	Sun et al. (2005)
Tetrandrine	S180 sarcoma (drug-resistant)	(Dosing not specified)	↓ MDR1; ↑ Fas & Trail; ↑ apoptosis in tumor tissues	Sun et al. (2005)

Because of its high invasiveness and heterogeneity, which can result in problems like metastasis and medication resistance, BC poses a serious threat to women all over the world. In clinical settings, the effectiveness of conventional therapeutic approaches in treating BC has been demonstrate to be limited. To stop the disease’s progression, more effective and focused treatment strategies must be developed immediately. Because of their extensive availability, varied biological activity, minimal toxicity and side effects, and good safety profiles, natural products and their derivatives have become attractive sources for the creation of new medications.

### 5.1 Technological advancements to increase plant metabolites’ efficacy

Recent technical breakthroughs have significantly improved the medicinal potential of plant-derived chemicals. Despite their potential biological activity, numerous endogenous metabolites are constrained by issues such as inadequate water solubility, insufficient bioavailability, and restricted target selectivity, which combined impede their therapeutic effectiveness. To tackle these issues, nanotechnology-based drug delivery methods have been created, including liposomes, solid lipid nanoparticles, polymeric nanoparticles, and micelles. These platforms enhance the solubility, stability, and targeted delivery of phytochemicals, thereby increasing their concentration at tumor locations while reducing systemic toxicity. For instance, arctigenin encapsulated in nanoliposomes has exhibited higher anti-tumor efficacy and increased bioavailability *in vivo*. Moreover, the advent of stimuli-responsive delivery systems and bioadhesive hydrogels facilitates controlled and prolonged release, hence enhancing therapeutic efficacy. Green synthesis methods, employing plant extracts for nanoparticle production, have garnered interest as biocompatible and environmentally sustainable alternatives to traditional fabrication techniques. These innovations collectively signify substantial advancement in the translational use of natural compounds in oncology, facilitating the development of more efficient and targeted cancer treatment strategies (Feng et al., 2017; Kiran et al., 2023; Kumar et al., 2021; Patra et al., 2018; Staes et al., 2019).

## 6 Traditional Chinese medicine (TCM) and herbal formulations in breast cancer therapy

Standard interventions including surgery, chemotherapy, radiation, targeted therapy, and endocrine therapy continue to be the principal methods for treating BC. Nonetheless, these methods frequently have significant restrictions, such as the dangers of metastasis, recurrence, postoperative difficulties, and other side effects (Waks and Winer, 2019). In recent years, TCM has garnered heightened interest as a supplemental and holistic supplement to conventional therapy. Grounded on ancient Chinese medical philosophy, Traditional Chinese Medicine employs a tailored, multi-faceted therapeutic approach that corresponds effectively with the intricate pathophysiology of cancer (Spronk et al., 2018). TCM is extensively utilized in China for cancer treatment, particularly BC, owing to its accessibility, cost-efficiency, and historical clinical application (Wang et al., 2018). Recent studies endorse its capacity to regulate immunological responses, inhibit tumor proliferation, and alleviate the adverse effects associated with standard therapies including chemotherapy and radiotherapy (Feng R. Q. et al., 2023). Consequently, although traditional therapies are fundamental to BC management, TCM is increasingly acknowledged as a significant element of comprehensive, long-term care programs. TCM exerts its anticancer effects through many methods, including immunological regulation, activation of tumor cell death, blockage of angiogenesis, and suppression of tumor cell proliferation (Xie et al., 2023). Its therapeutic repertoire encompasses a diverse array of historically employed botanical medicines, scientifically sanctioned plant metabolites, and recognized herbal formulations originating from the Chinese medicinal tradition. These agents establish a strong basis for their incorporation into integrative oncology, especially for BC. Several metabolites from traditional Chinese medicinal sources have shown promise anticancer action, including potential effectiveness against BC and TNBC. The chemicals and the supporting evidence are described in Table 3 (Li et al., 2025).

**TABLE 3:** Bioactive phytoconstituents targeting metastatic breast cancer.

Group name	Compound name	Biological name	Mechanism of action	References
Polyphenols	Curcumin	*Curcuma longa*	The initiation and articulation of eGFR are suppressed, leading to the induction of apoptosis. Furthermore, there is a concurrent inhibition of NF-κB/AP-1 and MAPK pathways, as well as a reduction in MMP-9 expression, which is typically stimulated by TPA.	Wu et al. (2021)
	Resveratrol	*Vitis vinifera*	Inhibition of Wnt β –β-catenin pathway	Fu et al. (2014)
	Eugenol	*Syzygium aromaticum*	Inhibition of NF-κB signalling reduces the production of IL-8 and IL-6	Islam et al. (2018)
	Oleuropein	*Olea europaea*	Inhibition of MCF-7 cell development	Sepporta et al. (2015)
	Gossypol	*Gossypium hirsutum*	Overexpression of BNIP3, TNFRSF9, and GADD45A over cell lines MDA-MB-231 and MDA-MB- 468	Messeha et al. (2019)
	Pterostilbene	*Cyanococcus*	Inhibition of mTOR and AKT phosphorylation, Suppression of cyclin D1 expression in MDA-MB 468 cell lines	Wakimoto et al. (2017)
Flavonoids	Nobiletin	*Citrus depressa*	Suppression of ERK1/2, cyclin-D1; over-expression of p21 Decrease in the activities of mTOR and AKT over the MCF-7 cell lines	Noguchi et al. (2016)
	Baicalein	*Scutellaria baicalensis*	Decrease in the NF-κB-p65 protein production; Raised expression of BCL2, BIRC3, BIRC2, and CCND1 over MCF-7 and MDA-MB-231cell lines	Gao et al. (2018)
	Ellagic acid	*Juglans regia*	Attenuation of TGF-β/Smads over MCF-7 cell lines	Chen et al. (2015b)
	Genistein	*Glycine max*	Downregulation of the Hedgehog-Gli1 Signaling over MCF-7 Cell lines	Chen and Chien. (2019)
	Kaempferol	*Moringa oleifera*	Prevention of oncogene transformation by production of NRF2 and its NQO1 enzyme in MCF-7 cells over MDA-MB231 cell lines	Akram et al. (2017)
	Myricetin	*Camellia Sinensis*	Downregulation of ST6GALNAC5 expression in MDA-MB-231 Cell lines	Ci et al. (2018)
Terpenoids	Astragaloside IV	*Astragalus membranaceus*	Inhibition of cell growth and metastasis by activating TRHDE-AS1 expression over cell lines: MCF-7, MDA-MB-231, and MDA-MB-468	Hu et al. (2021)
	Ursolic acid	*Ocimum tenuiflorum*	Downregulation of EGFR, PI3K/Akt/mTOR, and ERK over MDA-MB-231 cell lines	Yin et al. (2018)
	β-Elemene	*Rhizoma zedoariae*	Limiting aerobic glycolysis-driven nuclear transfer over MDA-MB-231 and MCF-7 cell lines	Pan et al. (2019)
	D Rhamnose β-hederin	*Clematis ganpiniana*	Blockage of PI3K/AKT pathway and stimulation of ERK pathway. Over MCF-7, MDA-MB-231, BT474, SUM1315 cell lines	Cheng et al. (2014)
	β-caryophyllene oxide	*Myrica rubra*	Suppression of NF-κB over cell lines: MDA-MB-231 and MCF7	Hanušová et al. (2017)
Alkaloids	Colchicine	*Colchicum autumnale*	By G2/M phase cell arrest	Sun et al. (2016)
	Cepharanthine	*Stephania cepharantha*	Disruption of AKT/mTOR system over MDAMB-231 and MCF-7 cell lines	Gao et al. (2017)
	Harmine	*Peganum harmala*	Via reduction in the expression of pErk, Bcl2, pAkt, and TAZ over MDA-MB-231 and MCF-7 cell lines	Ding et al. (2019)
	Lycorine	*Narcissus pseudonarcissus*	Disruption of Src/FAK pathway over MCF-7, T47D and MDAMB-231 cell lines	Ying et al. (2017)
	Noscapine	*Papaver somniferum*	Reduction in the expression of NF-κB gene and protein. Overexpression of IκBα gene over MCF-10F, MCF-7, MDA-MB-231 cell lines	Quisbert-Valenzuela and Calaf (2016)
	Piperlongumine	*Piper longum*	By decreasing Bcl-2, cyclin D1, p-Akt, p53, p70S6K1 and 4E-BP1 expression and over expression of Cytochrome C and Bax. By inhibition of PI3K/Akt/mTOR over MCF-7, MDA-MB-231, MDA-MB-453 and BT-549	Parama et al. (2021)
	Theacrine	*Theobroma grandiflorum*	Inhibition of TGF over MDA-MB-231 cell lines	Ko et al. (2019)

### 6.1 Evodiamine and rutaecarpine

Wu-Zhu-Yu, or Evodia rutaecarpa, has served as a fundamental element of TCM for generations. The fruit extract of this Rutaceae family plant comprises significant alkaloids, such as rutaecarpine and (±)-evodiamine (EVO). This plant, also referred to as Evodia officinalis Dode or Evodia rutaecarpa Bentham, remains extensively utilized in herbal compositions (Xiao et al., 2023). Evodiamine, a bioactive component, has demonstrated considerable anticancer efficacy in preclinical animals. Recent improvements in protein-based drug delivery technologies, specifically bovine serum albumin (BSA)-encapsulated nanoparticles, have enhanced the solubility, bioavailability, and targeted cytotoxicity of EVO in BC cells. BSA-based nanoparticles facilitate regulated drug release and improved apoptotic effects, indicating their prospective utility in precision oncology utilizing plant metabolites (Solanki et al., 2024). EVO has been demonstrated to inhibit topoisomerase I (TopI) by stabilizing the TopI-DNA covalent complex, hence obstructing DNA relaxing. In MCF-7 cells, EVO reduced TopI levels and elevated DNA-trapped complexes in a concentration- and time-dependent manner, underscoring its significance for anticancer drug development (Chan et al., 2009). Additionally, EVO-inspired polycyclic heterocyclic derivatives have been created with a scaffold-hopping technique to target TNBC, an aggressive subtype with restricted therapeutic alternatives. A lead chemical, identified as 7f, exhibited significant antiproliferative action (IC_50_ = 0.36 μM) in MDA-MB-231 cells through the formation of covalent TopI complexes and the induction of ROS-mediated DNA damage. This chemical shown activity in both *in vitro* and *in vivo* models of TNBC. EVO treatment at doses ranging from 5 to 40 µM for 48 h elicited ROS-mediated apoptosis in MDA-MB-231 cells. *In vivo*, intraperitoneal treatment of EVO at 5 mg/kg/day for 2 weeks in nude mice with TNBC xenografts resulted in a substantial decrease in tumor volume. Notwithstanding these findings, no clinical trials have been undertaken to yet (Xu X. et al., 2021; Yuan et al., 2022).

Rutaecarpine, an alkaloid derived from E. rutaecarpa, has demonstrated *in vitro* anticancer activity against MCF-7 and MDA-MB-231 cells at doses ranging from 10 to 50 µM during a duration of 48 h. It triggered apoptosis, as demonstrated by chromatin condensation and nuclear blebbing, and resulted in G_0_/G_1_ phase cell cycle arrest. The effects were more significant in MCF-7 cells, suggesting potential subtype-specific action (Cokluk et al., 2021). Evodiamine has demonstrated potential in surmounting drug resistance. In adriamycin-resistant NCI/ADR-RES BC cells, EVO suppressed proliferation with a GI_50_ value of 0.59 μM. It triggered apoptosis and G_2_/M phase cell cycle arrest, while also facilitating tubulin polymerization and the phosphorylation of Raf-1 at Ser338 and Bcl-2. *In vivo* studies indicated that EVO had greater antitumor activity than paclitaxel in xenograft models, implying promise for additional preclinical investigation against drug-resistant malignancies (Lei et al., 2022).

### 6.2 Icaritin

Epimedium, also referred to as Horny Goat Weed or Yin Yang Huo, is a traditional Chinese herb utilized for ages to address a range of diseases. The genus Epimedium, part of the Berberidaceae family, including species like Epimedium brevicornum, which possesses dried aerial portions abundant in bioactive chemicals, particularly icariin and its metabolite icaritin (IC) (Jin et al., 2019). In China, BC continues to be the primary cause of cancer-related mortality in women. Icaritin and its derivatives have attracted interest for their possible anti-tumor efficacy in preclinical animals. IC2, an icaritin derivative, has demonstrated the capacity to inhibit stearoyl-CoA desaturase-1 (SCD1), a lipogenic enzyme that is overexpressed in BC. This inhibition induces apoptosis and autophagy via the AMPK/MAPK pathway and reactive oxygen species (ROS) mediation (Yang et al., 2022; Wang Y. X. et al., 2023). The effects led to reduced proliferation and viability of MDA-MB-231 and MCF-7 cells in both *in vitro* and xenograft animal models. Nevertheless, clinical trials are required to validate these results. In MDA-MB-231 and MCF-7 cells, IC produced cell cycle arrest, reduced migration, and facilitated apoptosis through caspase-3 activation. In MCF-7 cells, it induced autophagy via the activation of the AMPK and ULK1 pathways. The anti-cancer efficiency of IC diminished in the presence of tamoxifen, whereas the suppression of autophagy augmented its activity—indicating that IC’s effectiveness may be dependent on estrogen receptors and vary by BC subtype (Tao et al., 2021). *In vitro* and *in vivo* models have shown the modulatory effects of IC on lipid metabolism and apoptosis at dosages of 1–20 µM over 24–72 h. In xenograft mice, daily oral dose of IC at 30 mg/kg for 21 days demonstrated positive anti-tumor effects; however, human clinical validation is forthcoming.

In the realm of TNBC, IC has demonstrated efficacy as a modulator of estrogen receptor alpha 36 (ER-α36), which facilitates estrogen signaling via the PI3K/AKT pathway. IC suppressed the proliferation of TNBC cells in both *in vitro* and *in vivo* experiments. The combination with cetuximab enhanced apoptosis and suppressed proliferation, demonstrating synergistic therapeutic potential. Furthermore, the co-administration of IC with the epigenetic inhibitor JQ1 demonstrated enhanced activity against treatment-resistant BC cells (Li et al., 2023). To enhance its distribution, poly (lactic-co-glycolic acid) (PLGA) nanoparticles modified with a hypoxia-cleavable RGD peptide (ARNP) were engineered to selectively target hypoxic BC tumors. These nanoparticles employed PEG cleavage and RGD–αvβ3 integrin binding to enable selective absorption by BC cells. In mouse models of bone metastases, ARNP significantly inhibited primary tumors and both bone and lung metastases, while also diminishing bone resorption (Li et al., 2023). Hydrous icaritin (HICT), a flavonoid derivative of IC, was engineered into nanorods (NRs) to mitigate its inadequate solubility. The HICT NRs demonstrated improved stability, elevated drug-loading capacity, and prolonged release characteristics. Both *in vitro* and *in vivo* investigations revealed enhanced anticancer activity, with results akin to paclitaxel, underscoring its potential for BC treatment (Wang Z. et al., 2020). IC has demonstrated potential as a radiosensitizer. In 4T1 BC cells, IC augmented the effects of ionizing radiation (IR) by diminishing cell proliferation, decreasing ERK1/2 and AKT activation, causing G_2_/M cell cycle arrest, boosting apoptosis, and lowering angiogenesis (Hong et al., 2013). IC functions as a dietary phytoestrogen, demonstrating dose-dependent effects. At low concentrations, it may promote BC cell proliferation; however, at elevated concentrations, it inhibits estradiol-induced growth by activating aryl hydrocarbon receptor (AhR) signaling, destabilizing ERα protein, and suppressing ERα-positive BC progression both *in vitro* and *in vivo* (Chen F. P. et al., 2015). These findings highlight the intricate pharmacodynamics of IC and the necessity for additional study to confirm its therapeutic function.

### 6.3 Matrine

Matrine (MAT) is a tetracyclic quinolizidine alkaloid extracted from the desiccated roots of Sophora flavescens Aiton, commonly referred to as Ku Shen in traditional Chinese medicine. It has showed a broad spectrum of pharmacological activity, including lipid-lowering, anti-inflammatory, anti-fibrotic, and anticancer properties (Zhou et al., 2021). In BC models, MAT has demonstrated the ability to limit MCF-7 cell proliferation, induce apoptosis and autophagy—evidenced by elevated LC3-II and reduced p62—and suppress AKT and mTOR phosphorylation, suggesting the involvement of the AKT/mTOR pathway in its mechanism of action (Du et al., 2020). Subsequent research has indicated that MAT suppresses EMT and tumor proliferation in both *in vitro* and *in vivo* settings. The effects were ascribed to the modification of the LINC01116/miR-9-5p/ITGB1 signaling axis, underscoring MAT’s potential significance in BC therapy (Ren et al., 2025). In TNBC, MAT inhibited the proliferation, invasion, and colony formation of MDA-MB-453 and HCC-1806 cells, while increasing apoptosis. *In vivo*, MAT decreased tumor development and HN1 expression, with these effects reversed by HN1 overexpression, indicating that HN1 may serve as a pivotal therapeutic target for MAT in TNBC (Guo et al., 2023; Wei et al., 2023).

Matrine has been assessed for its impact on multidrug-resistant BC cells. It suppressed the growth of MCF-7/ADR cells and mitigated resistance by altering the PI3K/AKT pathway, reducing the expression of P-glycoprotein (P-gp) and MRP1, while enhancing pro-apoptotic proteins (Zhou et al., 2018). In TNBC murine models, MAT delivered intraperitoneally at 25 mg/kg and *in vitro* at 1–10 mM for 48 h markedly diminished tumor volume and enhanced apoptosis, with PBS-treated subjects acting as controls (Du et al., 2020). In canine mammary carcinoma cells, MAT exhibited anticancer efficacy via promoting autophagy and downregulating BTF3, a protein discovered through a biotin-labeled probe. MAT also improved the thermal stability of BTF3, reinforcing its mechanistic significance (Feng Z. et al., 2023). Furthermore, a separate study indicated that MAT diminished BC cell viability and tumor proliferation by downregulating vascular endothelial growth factor (VEGF) and blocking the Wnt/β-catenin signaling pathway (Xiao et al., 2018). Simultaneously, sophisticated diagnostic techniques employing dendritic boronic acid-modified magnetic nanoparticles (MNPs) have been utilized to track circulating tumor cells (CTCs) in murine models of metastatic breast cancer. These instruments proficiently monitored therapeutic responses to MAT, doxorubicin, and their combination, offering a potential framework for real-time surveillance and individualized cancer treatment (Chen et al., 2024). These findings collectively underscore MAT’s diverse anticancer efficacy especially in BC and TNBC via mechanisms that encompass apoptosis, autophagy, EMT suppression, and the regulation of various signaling pathways. Nonetheless, clinical trials are required to confirm its safety and efficacy in human subjects.

### 6.4 Shikonin

Shikonin (SHK) is a bioactive naphthoquinone molecule extracted from Lithospermum erythrorhizon, commonly referred to as Zi Cao or purple gromwell in TCM. Besides its therapeutic applications, it has traditionally functioned as a natural color for fabrics and food. The herb, cultivated in China, Korea, and Japan (known as shikon), has been historically esteemed for its medicinal attributes (Wen et al., 2012). Shikonin exhibits a diverse array of biological actions, encompassing anti-tumor, anti-inflammatory, antioxidant, antibacterial, antiviral (particularly HIV-1 suppression), and wound-healing properties (Cör Andrejč et al., 2022). The effects have been confirmed in both *in vitro* and *in vivo* models, indicating possible uses in oncology and immune-related illnesses (Mukherjee et al., 2022; Boulos et al., 2019). In BC models, SHK has demonstrated encouraging preclinical efficacy. *In vitro*, it reduced 4T1 cell viability, increased reactive oxygen species (ROS), disrupted mitochondrial membrane potential, exposed calreticulin, and induced apoptosis. *In vivo*, SHK inhibited tumor proliferation, increased CD8^+^ T cell counts, and diminished regulatory T cell populations, suggesting its possible dual function in cytotoxic and immune-modulatory processes (Yu et al., 2024).

SHK, when integrated with chitosan-silver nanoparticles (Chi-Ag NPs), augmented tumor accumulation, elicited necroptotic immunogenic cell death, and suppressed tumor development and metastasis in TNBC models (Liang et al., 2024). *In vitro* investigations utilizing SHK at doses of 2.5–10 µM for 24–48 h have shown the activation of apoptosis, production of reactive oxygen species, and regulation of the immunological response. In xenograft-bearing mice, daily intraperitoneal injection of SHK at a dosage of 4 mg/kg for 10 days led to substantial tumor shrinkage (Chen et al., 2019). Notwithstanding these encouraging outcomes, SHK has yet to undergo assessment in clinical studies. SHK also suppresses the migration and invasion of BC cells through the miR-17-5p/PTEN/Akt signaling pathway (Bao et al., 2021). The SK/siTGF-β nanoparticle platform augments chemo-immunotherapy by facilitating immunogenic cell death, dendritic cell maturation, and cytotoxic T lymphocyte activation, while inhibiting TGF-β-mediated immunosuppression and epithelial-to-mesenchymal transition (Li et al., 2022). SHK has been found as a selective inhibitor of inosine 5′-monophosphate dehydrogenase 2 (IMPDH2), an enzyme that is overexpressed in TNBC. It suppresses the proliferation of TNBC cells in a dose-dependent manner, with its efficacy diminished by guanosine supplementation or IMPDH2 knockdown, hence reinforcing its potential as a target for purine metabolism in TNBC (Wang et al., 2021). SHK demonstrates synergistic effects when utilized in combination therapy. Co-administration with metformin has demonstrated the ability to promote apoptosis, block epithelial-to-mesenchymal transition (EMT), and improve chemosensitivity in BC models (Tabari et al., 2022). Additionally, ribitol has been shown to enhance the cytotoxic effects of SHK by interfering with cancer cell metabolism and regulating apoptotic and oncogenic pathways, presenting a possible strategy for overcoming drug resistance and suppressing tumor growth (Doddapaneni et al., 2025).

### 6.5 Osthole

Cnidium monnieri is commonly incorporated in numerous TCM formulations, such as Bushen Zhuanggu, BuGuZhi Wan, Wenshen Zhuanggu, and FaZhi Heidou. The Bushen Zhuanggu formulation has been utilized in clinical practice for several years as an adjuvant treatment for metastatic breast cancer (Wu et al., 2017). Osthole (OST), a bioactive coumarin derivative (7-methoxy-8-isopentenoxycoumarin) derived from Cnidium monnieri, has exhibited inhibitory actions against many cancer types, including breast, cervical, liver, and lung tumors, in addition to leukemia (Wu et al., 2017). In the realm of BC, OST demonstrates anti-tumor efficacy by influencing many signaling pathways. A primary mechanism entails the overexpression of guanine nucleotide-binding protein subunit gamma-7 (GNG7), a tumor suppressor whose diminished expression correlates with worse clinical outcomes. The suppression of GNG7 not only promotes the proliferation of BC cells but also reduces the effectiveness of OST, underscoring the significance of GNG7 in mediating its anticancer properties (Mei et al., 2021). Moreover, OST has demonstrated the ability to limit metastasis in breast cancer by downregulating integrins ITGα3 and ITGβ5 and obstructing the focal adhesion kinase (FAK)/Src/Rac1 signaling pathway. The findings indicate that OST may be a viable choice for targeting metastatic breast cancer via both proliferation-inhibitory and anti-metastatic pathways (Chen et al., 2022).

### 6.6 Arctigenin

Arctigenin (ATG) is a dibenzyl butyrolactone lignan and the primary bioactive metabolite derived from the medicinal plant Arctium lappa. It has exhibited extensive pharmacological promise in addressing numerous human ailments, including cancer, immune-related disorders, and chronic diseases (Wang Z. et al., 2023; Wang et al., 2023c). BC continues to be a predominant cause of cancer-related death in women, and ATG has arisen as a compound of significance in this regard. Preclinical studies demonstrate that ATG inhibits proliferation, migration, invasion, and EMT in BC cells, principally by downregulating eukaryotic translation initiation factor 4E-binding protein 1 (4EBP1) (Luo et al., 2021). Moreover, in estrogen receptor-positive BC cells, ATG inhibits tumor proliferation by facilitating GSK3-mediated degradation of cyclin D1 and inducing cell cycle arrest, indicating its promise as a multi-targeted therapeutic treatment for BC (Zhu et al., 2020). *In vitro*, ATG has demonstrated dose- and time-dependent anti-proliferative effects at doses between 10 and 50 µM for a duration of 24–72 h. Limited *in vivo* efficacy has been found after intraperitoneal injection at 10 mg/kg in murine models (Feng et al., 2017). Nonetheless, its clinical efficacy is constrained by inadequate water solubility and fast metabolic breakdown. To overcome these constraints, sialic acid-functionalized nanoliposomes (SA-Lip) have been engineered to improve the stability and distribution of ATG. These specialized nanocarriers provide substantial ATG loading, augmented cellular absorption, and increased cytotoxicity, while reducing immune-related adverse effects. SA-Lip systems exhibit enhanced therapeutic efficacy in preclinical BC animals compared to traditional formulations (Liu et al., 2024). Notwithstanding these encouraging advancements, additional clinical research is requisite to assess the safety and usefulness of ATG in humans.

## 7 Novel functional foods, nutraceuticals, and drug delivery strategies

Metastatic breast cancer presents a substantial clinical challenge owing to its aggressive nature, resistance to standard medicines, and the notable adverse effects linked to existing treatment methods. The illness frequently arises from ductal hyperplasia, advancing from benign tumors to aggressive and metastatic phases due to the impact of numerous carcinogens. Metastatic cancer cells can perform both symmetric and asymmetric cell division, which contributes to tumor heterogeneity and resistance to treatment. In recent years, functional foods and nutraceuticals sourced from medicinal and health-enhancing plants have garnered interest as potential complements to conventional cancer treatments. These therapies provide many modes of action and may enhance treatment outcomes or diminish toxicity. Functional foods are abundant in bioactive metabolites, such as vitamins, alkaloids, flavonoids, terpenoids, and polyphenols, which have exhibited diverse anticancer activities (Alharbi et al., 2022; Graidist et al., 2015).

Phytochemicals present in these compounds have demonstrated efficacy in cancer prevention and suppression by reducing cell proliferation, inducing apoptosis, and downregulating estrogen receptor α (ER-α) expression. Furthermore, they regulate numerous essential signaling pathways implicated in tumor growth and stem cell preservation, including Hedgehog, NF-κB, Notch, and Wnt/β-catenin. These pathways are especially pertinent to the self-renewal and metastatic capabilities of BC stem cells. Approximately 70% of cancer-related fatalities are attributed to modifiable variables, including food and lifestyle. Nutraceuticals have been shown to augment immunological responses in MBC patients, including elevated tumor necrosis factor (TNF) production and enhanced natural killer (NK) cell activity (Guerra et al., 2021; Hu et al., 2019). These findings endorse further exploration of dietary modifications as adjunctive therapies in the management of metastatic breast cancer.

### 7.1 The role of health food plants in functional foods and nutraceuticals

Nutraceutical oncology pertains to the utilization of functional foods and nutraceuticals in the prevention, management, and supportive treatment of cancer. The phytochemical constituents in plant-based functional meals may produce additive or synergistic anti-tumor actions, perhaps exceeding the effectiveness of individual metabolites. These combinations have demonstrated potential in inhibiting cancer cell proliferation and may potentially diminish the probability of resistance emergence. Individuals diagnosed with BC may experience improved outcomes by adopting dietary patterns that limit simple carbohydrates and include moderate amounts of high-quality proteins, fiber, and healthy fats, especially those abundant in omega-3 fatty acids (Varzakas et al., 2016). Multiple preclinical studies have investigated the influence of particular nutraceuticals on cancer-related signaling pathways, establishing a foundation for subsequent research with genetically modified animal models and, ultimately, clinical trials. In addition to their active role in controlling disease progression, functional foods and nutraceuticals are being investigated as supplementary therapy to improve the quality of life for BC patients. Dietary therapies may provide protective benefits against the negative effects of chemotherapy and radiotherapy, including fatigue, gastrointestinal distress, and immunological suppression (Calvani et al., 2020; Di Napoli et al., 2023).

A new position paper by the Academy of Nutrition and Dietetics highlights the developing notion of functional foods, characterizing them as foods that provide health advantages beyond fundamental nutrition. This extensive category encompasses naturally nutrient-dense foods including fish, legumes, whole grains, and nuts, in addition to fortified and supplemented goods. This approach posits that even widely consumed foods, such as enriched white bread, can be deemed functional when supplemented with particular nutrients. Significantly, only a limited number of categories—such as sugar-sweetened beverages and alcoholic beverages—are generally excluded (Ellis, 2025). This evolving concept reflects a growing convergence between traditional foodstuffs, pharmaceutical agents, and even medical technologies, increasingly blurring the boundaries between these categories. As food science continues to evolve, functional foods-whether processed or unprocessed-are broadly categorized into three groups.

• Fortified foods designed to address nutrient deficiencies and related diseases;
• Probiotic-rich foods containing beneficial microorganisms to support gut microbiota balance;
• Metabolically active foods that influence enzymatic pathways, including genetically modified options, to maintain physiological functions.

Nutraceuticals, a term introduced by Dr. Stephen De Felice in 1989, refer to bioactive substances sourced from food that offer health advantages beyond fundamental nutrition, encompassing illness prevention and therapy (Puri et al., 2022). These may encompass isolated nutrients, botanical extracts, and concentrated dietary components, typically made into capsules, pills, or liquids for therapeutic application. Typical instances encompass dietary fibers for digestive and cardiovascular wellbeing; fatty acids and phytoestrogens for cognitive and immunological efficacy; probiotics for gastrointestinal equilibrium; and vitamins and minerals for metabolic and skeletal integrity. Novel functional food constituents, including carotenoids, isothiocyanates, phytoesterols, polyphenols, and flavonoids, are increasingly recognized for their antioxidant, anti-inflammatory, antibacterial, and antimutagenic attributes in various biological systems (Bagchi et al., 2015). Some of the functional foods and nutraceuticals involved in disease prevention and management has been summarize in the Table 4 given below.

**TABLE 4:** Health benefits of functional foods and nutraceuticals.

Functional Foods/Nutraceuticals	Bioactive compound	Target	Disease prevention	References
MUFA/PUFA	Eicosapentaenoic acid (EPA) and docasahexaenoic acid (DHA)	Lowers Blood glucose levels and triglycerides Prevents ischaemic stroke	Cardio Vascular Diseases	Banaszak et al. (2024)
Aloe vera	Aloe-emodin, aloin, aloesin, emodin, and acemannan	—	Acute dermatitis Irritable bowel syndrome	Conte et al. (2019), Ozogul et al. (2021)
Ginseng	Ginsenosides	—	Psychomotor functions, Skin homeostasis	Puri et al. (2022)
green tea	Catechin and epigallocatechin gallate (ECGC)	Suppress oxidative stress, inflammation, and cell death via activation of the nuclear factor erythroid 2-related factor 2 (Nrf2) pathway	Type-2 Diabetes Inflamation	Alkhatib et al. (2017)
Isoflavones	Genistein, daidzein, and glycitein	—	cancer, diabetes, cardiovascular diseases	Das et al. (2020)
Probiotics	Lactococcus and Bifidobacterium	Short chain fatty acid production, lowering of gut pH, and nutrient competition to stimulation of mucosal barrier function and immunomodulation	Diarrhoea, Allergic Reactions	Latif et al. (2023)

### 7.2 Role of novel drug delivery in metastatic breast cancer

Novel drug delivery systems (NDDS) are becoming increasingly vital in the management of MBC by improving medication targeting, reducing systemic toxicity, and combating multidrug resistance. Nanotechnology has emerged as a possible method for enhancing the targeted delivery of treatments directly to malignancies. Integrating nanotechnology with superior biomaterial engineering enables these systems to maximize drug distribution, prolong circulation duration, and enhance combination therapy techniques. Targeting tactics encompass passive targeting through the increased permeability and retention (EPR) effect and active targeting via ligand-receptor interactions, exemplified by CD44, which is overexpressed on numerous cancer cell surfaces (Khan et al., 2025; Mitra et al., 2015; Zhu et al., 2021).

Although the advantages of bioactive metabolites in cancer prevention and treatment are acknowledged, preserving their structural integrity throughout processing, storage, and administration poses a considerable problem. Ongoing research focuses on ensuring the regulated release of substances at designated target areas while maintaining sensory and nutritional quality. Nanotechnology has demonstrated significant efficacy in encapsulating, stabilizing, and delivering these molecules, hence enhancing their bioavailability and therapeutic potential (Otchere et al., 2023). Diverse nanoformulations, such as nanoencapsulation, have been created. This procedure involves encasing core elements within a solid or liquid matrix, leading to the formation of structures including nanocapsules, nanospheres, and solid-lipid nanoparticles (SLNs). Nanocapsules are vesicular systems that encapsulate a liquid core, whereas nanospheres consist of a bioactive substance uniformly distributed within a matrix. Nanosystems often measure under 1000 nm, with regulatory standards frequently necessitating particles smaller than 100 nm for medicinal and cosmetic uses. Targeted distribution to specific organs is generally accomplished with particles sized between 10 and 100 nm (Rehman et al., 2019).

Polymer-based nanodrug delivery systems have gained importance due to their versatility and compatibility with bioactive chemicals (Assadpour and Jafari, 2019). Prevalent encapsulating substances comprises: carbohydrate includes starch, cellulose derivatives, pectin, alginate, carrageenan, chitosan, and gums such as gum arabic, which are esteemed for their matrix-forming, emulsifying, and film-forming properties (Tabassum et al., 2023). Proteins such as soy protein, gelatin, casein, and whey, which offer amphiphilic properties and strong binding to both hydrophilic and hydrophobic compounds; Lipids, encompassing natural fats, mono- and diglycerides, phospholipids, and waxes, are proficient at encapsulating hydrophobic molecules such as aromatic or volatile chemicals owing to their moisture barrier characteristics.

Nano-delivery technologies have effectively encapsulated various nutrients and bioactives in food and medicinal applications. This encompasses vitamins (A, D, E) in beverages; calcium in baked goods; carotenoids (β-carotene, lutein, lycopene, astaxanthin) in dairy products; polyphenols (quercetin, resveratrol, catechins) in yogurt and oils; and antioxidants (α-lipoic acid, α-tocopherol) in fruit juices (Otchere et al., 2023). Polysaccharide-based systems are recognized for their bioadhesive characteristics, whereas lipid-based nanodrug delivery systems—such as nanoliposomes, solid lipid nanoparticles (SLNs), and nanostructured lipid carriers (NLCs)—provide biocompatibility, elevated loading efficiency, and enhanced solubility and bioavailability. Additional delivery platforms include nanoemulsions, nanogels, and Pickering emulsions (a subtype of NLCs), which are being increasingly explored for both food-grade and pharmaceutical applications (Livney, 2015; McClements and Öztürk, 2021; Hu et al., 2017). Collectively, these technologies underscore the growing significance of NDDS in enhancing the transport and effectiveness of bioactive chemicals in oncology and other fields.

### 7.3 Advancements in novel drug delivery systems for metastatic breast cancer

Nanoparticle-based drug delivery systems have markedly enhanced the therapeutic efficacy of several bioactive natural substances in BC treatment, particularly by augmenting their bioavailability, cellular absorption, and sustained release characteristics. Curcumin-albumin nanoparticles were effectively produced and exhibited enhanced antiproliferative activity relative to free curcumin in MDA-MB-231 cells. In rat models, the nanoparticulate formulation demonstrated superior tissue targeting, pharmacokinetics, and prolonged drug release, suggesting better chemotherapeutic efficacy (Zhou et al., 2024). Resveratrol-loaded solid lipid nanoparticles (Res-SLN) demonstrated superior reduction of proliferation, invasion, and migration of MDA-MB-231 cells compared to free resveratrol, indicating its potential application in BC management (Wang et al., 2017). Sabapati et al. (2019) synthesized Annona muricata fruit extract into solid lipid nanoparticles (SLNs), which exhibited increased cytotoxicity and apoptotic activity in MCF-7 cells relative to the unformulated extract. This suggests a possible approach to diminish adverse effects while enhancing therapeutic efficacy. Pillai et al. (2020) presented a BioPerine-encapsulated chitosan (CS)-PEG-PLA hybrid nanoparticle, sourced from black pepper alkaloids. These nanoparticles demonstrated considerable *in vitro* cytotoxicity against MDA-MB-453 cells and more efficiently downregulated P-glycoprotein (P-gp) expression than the commercial inhibitor verapamil hydrochloride, underscoring their potential to surmount multidrug resistance. Sezer (2021) created silymarin-loaded solid lipid nanoparticles (SLNs) that exhibited increased apoptotic rates and proliferation suppression in A549 and MCF-7 cells relative to free silymarin, therefore addressing its solubility constraints. Gilani et al. (2021) developed luteolin-encapsulated chitosan-coated nanostructured lipid carriers (Ch-NLCs), demonstrating dose- and time-dependent cytotoxicity and superior antioxidant activity against MDA-MB-231 and MCF-7 cells, surpassing uncoated or pure luteolin.

Ellagic acid (EGA) was incorporated into an apamin-mediated lipidic nanoemulsion (EGA-EML-APA), exhibiting enhanced cytotoxicity (IC_50_ = 5.47 μg/mL) and inducing G2/M and S-phase arrest in MCF-7 cells, in contrast to free ellagic acid (Badr-Eldin et al., 2022). Truong et al. (2022) formulated tetrahydrocurcumin (THC)-encapsulated Ch-NLCs, improving *in vitro* dermal penetration, cellular absorption, and cytotoxicity in MDA-MB-231 cells. These carriers demonstrated potential for transdermal administration in the treatment of TNBC. Emami et al. (2023) developed polyphyllin D-loaded solid lipid nanoparticles (SLNs), which exhibited significant cytotoxic effects in MCF-7 and MDA-MB-231 cells (IC_50_ values of 33.25 and 35.74 μg/mL, respectively), along with good *in vivo* efficacy in BALB/c mice and no observable toxicity. Wardana et al. (2023) produced nanoencapsulated Uncaria gambir as a chemopreventive agent. The nanoformulation improved both *in vitro* and *in vivo* efficacy against BC, surpassing the free extract in therapeutic effectiveness. These results collectively underscore the significance of nanotechnology in enhancing the solubility, stability, cellular targeting, and therapeutic efficacy of plant-derived bioactive chemicals in the treatment of BC. Table 5 presents a summary of these formulations and their preclinical findings.

**TABLE 5:** Patented phyto-novel drug delivery formulations in metastatic breast cancer.

Date of publication	Patent number	Title	Patent description	References
24.03.2005	US20070259060	Formulations and methods for treating BC with *Morinda citrifolia* and Methylsulfonymethane	This patent showed the latest methods for treating and preventing early-stage mammary BC by providing a safe, nutraceutical formulation of *Morinda citrifolia*, Methylsulfonylmethane (MSM), and other substances	Mian-Ying et al. (2007)
14.11.2014	KR1020160057716	Pharmaceutical composition for preventing and treating BC, comprising quercetin	This patent describes a BC prevention and therapy drug including quercetin or a methoxy polyethylene glycol-polylactide-quercetin nanoparticle. Injecting quercetin, a bioflavonoid, with an MPEG-PLA-Qu nanoparticle produced by encapsulating it in an MPEG-PLA nanoformulation suppresses cancer tissue growth and induces apoptosis in BC cells	Lee et al. (2016)
17.07.2015	IN781/KOL/2015	A process of preparing efficient herbal nanoparticles of Solasodine for BC	This patent claims to generate herbal solasodine nanoparticles using gelatin type B, PMMA, toluene, and glutaraldehyde in microemulsion cross-linking. To treat BC, solasodine herbal nanoparticles are delivered to the therapy site	Bhattacharya et al. (2015)
15.01.2018	IN201841001612	Formulation of Allium sativum and Murraya koenigii based phytosomal complex for the sustained release and treatment of BC using phospholipids from milk	According to this patent, a phytosome including extracts of Murraya koenigii and Allium sativum and a natural phospholipid extracted from milk can be used to prevent or treat prostate cancer or to stop BC from returning after treatment	Azeezn et al. (2018)
26.09.2019	WO2018067570	Mangiferin encapsulated gold nanoparticles, fabrication methods and cancer therapeutic methods	This study created encapsulated gold nanoparticles by mixing Mangiferin with reducing chemicals in a liquid media. Gold salts are added to the reducing agent. A solution of stabilized, biocompatible gold nanoparticles coated with mangiferin is formed when the gold salts react without a reducing agent. Radioactive gold salts and AuCl4 are gold salts used	Kattesh et al. (2018)
02.12.2019	CN110917139	Preparation and application of multi-branch biotin modified BC targeted liposomes	The invention reveals new lipid materials for BC drug delivery. Polyethylene glycol-extended cholesterol and BC-preventing biotins are connected by a branch structure in the novel lipid materials. Novel lipid materials’ affinity for receptors can improve tumor targeting and BC treatment. Paclitaxel-loaded liposomes target breast cancer and have many uses. Liposomal, nanoparticle, and micelle dosage forms can use the unique lipid materials	Yong et al. (2019)
16.01.2020	CN111249254	Preparation method and application of folic acid coupling albumin nanoparticles loaded with Baicalin	This patent addresses producing and using baicalin-loaded folic acid coupling albumin nanoparticles. This increases baicalin’s biological availability and nanoparticles’ tumor targeting and anticancer activity. Nanoparticles have several uses, including making BC drugs, and are non-toxic	Yu et al. (2020b)
12.08.2021	AU2021105352	Green synthesis of Silver Nanoparticles with Prunus cerasoides extracts as a potential Hepatic and BC Agent	According to this patent, Prunus cerasoides extracts were utilized to produce silver nanoparticles (AgNPs) and test their cytotoxicity against breast and liver cancer cell lines. The current study found that green-produced AgNPs are highly cytotoxic against HepG 2 and MCF-7 cell lines, suggesting they could be employed to treat breast and liver cancer	Khan et al. (2021)
15.03.2023	CN116370436	Application of emodin polymer lipid hybrid nanoparticles	This patent describes emodin polymer lipid hybrid nanoparticles. An IL-6/JAK2/STAT3 signal channel inhibitor uses emodin polymer lipid hybrid nanoparticles. Emodin polymer lipid hybrid nanoparticles are made from polylactic acid-glycolic acid copolymer, lipid material, and distearoyl phosphatidyl ethanolamine-polyethylene glycol 2000	Yu et al. (2023)
13.06.2024	CN118750450	Preparation method and application of rhizome Atractylodis macrocephalae extract self-assembled carrier-free nanoparticles	This patent shows how to make and use bighead Atractylodes Rhizome extract self-assembled carrier-free nanoparticles. The self-assembled carrier-free nanoparticles show that nanotechnology enhances the insoluble components of a bighead Atractylodes Rhizome extract	Jin et al., 2024

## 8 Mechanistic insights and future perspectives

Cancer is a multifactorial illness governed by an intricate network of signaling channels that regulate the expression of genes associated with cell proliferation, survival, migration, and metastasis. The Signal Transducer and Activator of Transcription 3 (STAT3) signaling cascade is a crucial route that regulates angiogenesis, invasion, migration, and unchecked cell proliferation (Yuan et al., 2015). Constitutive activation of STAT3 has been documented in multiple cancer types, including BC, and is especially common in TNBC (Banerjee and Resat, 2016). STAT3 propels tumor advancement via the transcriptional activation of genes pertinent to cell cycle regulation (e.g., c-Fos, MEK5, c-Myc), apoptosis suppression (survivin, Mcl-1, Bcl-xL), and angiogenesis (VEGF, COX-2, MMP-2) (Hsieh et al., 2005). Increased STAT3 activity in TNBC enhances tumor aggressiveness, EMT, and metastatic potential. Numerous phytochemicals have exhibited anticancer properties by inhibiting STAT3 signaling and its downstream effectors. Mistletoe (Viscum album L.) extract decreased STAT3 signaling, resulting in the downregulation of survivin, matrix metalloproteinases (MMPs), and EMT markers, which combined triggered apoptosis, lowered tumor development, and diminished lung metastasis *in vivo* (Kwon et al., 2021). Likewise, Centipeda minima extract demonstrated anti-metastatic properties in MDA-MB-231 cells by suppressing STAT3 and related pathways (Lee et al., 2020). Moreover, extracts from Origanum syriacum and Elaeagnus angustifolia prompted apoptosis in TNBC cells by concurrently suppressing STAT3 and activating the tumor suppressor protein p53 (Fouzat et al., 2022).

Metastasis is a complex and coordinated multi-step process that encompasses intrinsic modifications in cancer cells as well as changes in the tumor microenvironment. EMT is a fundamental process that facilitates cancer spread. While EMT serves a physiological function in organogenesis, embryonic development, and wound healing, its dysregulated activation in cancer results in the conversion of adherent, polarized epithelial cells into motile, invasive, and non-polar mesenchymal cells capable of metastasis (Singh et al., 2018). During EMT, epithelial markers like E-cadherin and claudin are downregulated, whilst mesenchymal markers such as vimentin and N-cadherin are increased. The phenotypic flip is regulated by a group of transcription factors termed EMT-activating transcription factors (EMT-TFs), which encompass members of the Snail, Slug, ZEB, and Twist families. These EMT-TFs inhibit E-cadherin expression and promote cellular reprogramming. EMT is regulated by multiple critical signaling pathways, specifically Wnt/β-catenin, TGF-β, Hedgehog (Hh), and Notch pathways, which collectively influence the invasive and metastatic characteristics of cancer cells (Majidpoor and Mortezaee, 2021).

## 9 Critical evaluation and future directions

This study includes a wide range of plant-derived chemicals and their potential benefits against metastatic breast cancer. However, the majority of the supporting data comes from preclinical, *in vitro* studies. Although these findings are promising, they are currently insufficient for therapeutic use. A careful examination of the literature reveals several enduring limitations that hinder repeatability, including differences in plant extract production, dosage selection, and test conditions. It may be difficult to accurately attribute reported effects to the test substances since many studies lack adequate experimental controls. Furthermore, most research is limited to short-term cell-based models that are unable to adequately represent the complexity of the tumor microenvironment. Promising compounds often have poor solubility or stability, even though their pharmacokinetic or bioavailability profiles are not assessed. Crucially, there is a significant translational gap due to the infrequent investigation of therapeutic indices, drug–herb interactions, and long-term toxicity.

Moreover, there are regulatory loopholes pertaining to the categorization, authorization, and marketing of botanical medications and nutraceuticals. Clinical adoption is delayed by the current frameworks’ frequent absence of explicit instructions for multi-component natural formulations. Bioavailability is another significant obstacle; many drugs have poor solubility and fast metabolism, which restricts their systemic exposure. Despite offering promising answers, innovative delivery technologies like liposomes and nanoparticles are still in the early stages of clinical practice integration. Future studies should focus on comprehensive *in vivo* validation using therapeutically relevant animal models, dose-response studies, mechanistic investigations, and the development of nanotechnology-based delivery systems in order to increase bioavailability and target specificity. Furthermore, to ensure taxonomic accuracy and reproducibility, well-established methods such as ConPhyMP and MPNS must be applied. These issues must be address in order to translate laboratory data into safe, effective, and realistically applicable treatments for metastatic breast cancer. Overcoming these scientific, technical, and regulatory obstacles is crucial for converting preclinical discoveries into safe, effective, and clinically applicable treatments for metastatic breast cancer care.

## 10 Conclusion

Metastatic breast cancer, especially the triple-negative kind, is among the most aggressive and challenging cancers to treat, highlighting the pressing necessity for innovative therapeutic approaches. This review emphasizes the potential function of functional foods and plant-derived metabolites as adjunctive agents that may influence critical molecular pathways associated with cancer progression, including reactive oxygen species (ROS)-mediated apoptosis, epithelial–mesenchymal transition (EMT), and STAT3 signaling. When combined with advancements in targeted drug delivery methods and nanotechnology, these natural chemicals present significant opportunities to improve therapeutic efficacy while reducing systemic toxicity. Nonetheless, it is crucial to acknowledge that most supporting evidence is preclinical, necessitating comprehensive clinical trials to validate these discoveries and evaluate their safety, effectiveness, and long-term effects in human populations. Incorporating nutraceuticals and botanical agents into a holistic cancer therapy strategy has significant promise to enhance patient quality of life, address drug resistance, and aid in the prolonged control of metastatic breast cancer. Ongoing interdisciplinary research is crucial for converting these promising natural interventions into clinically applicable therapeutics.
